# Correspondence

**Published:** 1875-10

**Authors:** 


### Correspondence.

*Editors Buffalo Medical and Surgical Journal:*

GENTLEMEN—I desire to call your attention, and through you the attention of the profession, to a matter of interest in *Life Insurance.* Life insurance agents are often applying to physicians for certificates of the good health of patients.

This is one of the most delicate and dangerous procedures for a physician, and I desire hereby to dissuade all physicians from ever doing anything of the sort, unless employed by the company in a manner wholly independent of agent and patient. The knowledge a physician has of his patient is the most valuable an insurance company *can* possess—worth ten times as much as what they can possibly obtain through any physical examination by their examining surgeon. It is not over safe for an attending physician to tell what he knows of history and habits, and I have in my life by so doing lost the patronage of one of the best paying families in the city.

Life insurance companies are mean and small enough to ask this without in any way offering compensation; and my brief but earnest advice is, never make out family physician's certificate for patient or agent. If you consent to do it at all, do it unbeknown to patient or agent, for the company, and in consideration of adequate compensation. Yours very truly,

ONE WHO KNOWS.

